# Serotype and multilocus sequence typing of *Streptococcus suis* from diseased pigs in Taiwan

**DOI:** 10.1038/s41598-023-33778-9

**Published:** 2023-05-22

**Authors:** Ching-Fen Wu, Siou-Hui Chen, Chi-Chung Chou, Chao-Min Wang, Szu-Wei Huang, Hung-Chih Kuo

**Affiliations:** 1 Department of Veterinary Medicine, National Chiayi University, Chiayi City, Taiwan; 2 Department of Veterinary Medicine, National Chung Hsing University, Taichung City, Taiwan

**Keywords:** Antimicrobials, Bacteria

## Abstract

*Streptococcus suis* (*S. suis*) infection can cause clinically severe meningitis, arthritis, pneumonia and septicemia in pigs. To date, studies on the serotypes, genotypes and antimicrobial susceptibility of *S. suis* in affected pigs in Taiwan are rare. In this study, we comprehensively characterized 388 *S. suis* isolates from 355 diseased pigs in Taiwan. The most prevalent serotypes of *S. suis* were serotypes 3, 7 and 8. Multilocus sequence typing (MLST) revealed 22 novel sequence types (STs) including ST1831-1852 and one new clonal complex (CC), CC1832. The identified genotypes mainly belonged to ST27, ST94 and ST1831, and CC27 and CC1832 were the main clusters. These clinical isolates were highly susceptible to ceftiofur, cefazolin, trimethoprim/sulfamethoxazole and gentamicin. The bacteria were prone to be isolated from cerebrospinal fluid and synovial fluid in suckling pigs with the majority belonging to serotype 1 and ST1. In contrast, ST28 strains that corresponded to serotypes 2 and 1/2 were more likely to exist in the lungs of growing-finishing pigs, which posted a higher risk for food safety and public health. This study provided the genetic characterization, serotyping and the most current epidemiological features of *S. suis* in Taiwan, which should afford a better preventative and treatment strategy of *S. suis* infection in pigs of different production stages.

## Introduction

*Streptococcus suis* (*S. suis*), a gram-positive coccus with capsule, is one of the most important pathogens in the swine industry. As an opportunistic pathogen, *S. suis* colonizes the upper respiratory tract (tonsil and nasal cavity) of the pigs. The infected pigs commonly manifest meningitis, arthritis, pneumonia, septicemia, and may be accompanied by acute death^1^. The capsular polysaccharides (CPSs) antigen of the bacteria is used as the basis of classification and 29 serotypes have been identified by using polymerase chain reaction (PCR)^2^. Notably, *S. suis* is a zoonotic bacterial pathogen that endangers the people in close contact with the infected pigs or pork products. Among the identified serotypes, serotype 2 is considered the most prevalent and virulent serotype in pigs and humans^1^.

Different serotypes of *S. suis* can be isolated in the same herd, which suggests that similar clinical symptoms may not be caused by *S. suis* strains with identical serotypes. In addition, the importance of specific serotypes may vary geographically. Traditional typing methods only identify the strains that are single serotype or represent specific geographic locations, which renders the findings difficult to be compared across laboratories. Therefore, in comparison with the coagglutination test, genetic typing methods that can be used to rapidly screen the bacterial isolates with lower cost are favorable for the analysis of colonial structure and genetic diversity of *S. suis*. The knowledge is eminent to the understanding of the epidemiology of *S. suis* and to identify specific strains with high pathogenicity, which could ultimately contribute to the prevention of the disease progression^2^. Multilocus sequence typing (MLST) has been successfully used in many studies of molecular epidemiology in bacteria. It has been used for identifying the *S. suis* genotypes in order to confirm the differences between the sequence types (STs) and ST clonal complexes (CCs) of *S. suis* strains ^3^.

Among the identified *S. suis* serotypes, serotype 1 is related to the neurological symptoms accompanied by cerebral microlesion, while serotypes 2–8 tend to cause pulmonary lesions^4^. In comparison to the *S. suis* strains that only cause pneumonia, the strains causing meningitis, septicemia and arthritis are more likely to exhibit typical clinical symptoms, which may be attributed to the pathogenesis of different serotypes^5^. Different strains also exert significant differences in drug resistance. The *S. suis* strains isolated from clinical cases of pigs are less resistant to ampicillin, ceftiofur, enrofloxacin, florfenicol, penicillin, and sulfamethoxazole-trimethoprim, whereas a high proportion of the strains is resistant to tetracycline^6^. However, the strains isolated from the tonsils of healthy animals or the environment of the butcheries are usually multi-drug resistant^7,8^. In this study, we focus on the epidemiology and laboratory findings of clinical isolates of *S. suis* in diseased pigs in Taiwan, investigating the association among the distribution, serotypes, genotypes and the antimicrobial drug susceptibility.

## Materials and methods

### Ethical statement

All the diseased pigs were sent to Animal Disease Diagnostic Center in National Chiayi University by the farm owners for necropsy. Sacrifice of the diseased pigs, tissue collection and further bacterial isolation and identification were essential in the standard process for disease diagnosis and treatment. This study project collected the deposited samples for in-depth analysis. The Institutional Animal Care and Use Committee (IACUC) confirmed that this project did not involve animal experiments and approval of animal use protocol was not required.

### Isolation and identification of isolates

From March 2017 to October 2021, a total of 388 isolates from 355 diseased pigs in 289 infected herds submitted to the Animal Disease Diagnostic Center (ADDC), Department of Veterinary Medicine of National Chiayi University (Taiwan) for necropsy were collected for analysis. All of the specimens, including liver, lung and bronchus, as well as the cerebrospinal fluid and synovial fluid, were aseptically sampled during necropsy. The specimens were cultured on a 5% defibrinated sheep blood agar plate (BBL*™* Blood Agar Base, Infusion Agar, BD, USA) and incubated at 37 °C for 18–24 h. The *S. suis* isolates were suspected when α hemolytic colonies were observed. Bacterial DNA was extracted from the *S. suis*-suspected colony using the Taco™ DNA/RNA Extraction Kit (Taco, Taiwan). The primer pair designed for the *gdh* gene of *S. suis* were used for PCR^9^. The PCR products of positive samples were sequenced in both directions by Tri-I Biotech Inc (Taipei, Taiwan) and the sequences were analyzed and compared using the basic local alignment search tool (BLAST) database on the National Center for Biotechnology Information (NCBI) website. After identification, bacteria were stored in brain heart infusion broth (BHIB) containing 10% fetal calf serum (FCS) and 20% glycerol at − 80 °C.

### Serotyping by multiplex PCR and restriction fragment length polymorphism

The primer pairs designed for *cps* genes for multiplex PCR^10^ were used to categorize the *S. suis* isolates into group 1 (serotypes 1/2, 1, 2, 3, 7, 9, 11, 14, and 16), group 2 (serotypes 4, 5, 8, 12, 18, 19, 24, and 25), group 3 (serotypes 6, 10, 13, 15, 17, 23, and 31) and group 4 (serotypes 21, 27, 28, 29, and 30). The PCR products were sequenced, analyzed and compared with NCBI BLAST database for the confirmation of *S. suis* serotypes. In addition, PCR-restriction fragment length polymorphysim (RFLP) was performed for the *S. suis* isolates that were temporarily identified as serotypes 1, 1/2, 2 and 14. The *cpsK* gene of these isolates were amplified by PCR and the PCR products were cleaved by restriction endonuclease *Bst*NI (NEB®, USA) at 60 °C for 1 h. The PCR–RFLP products were analyzed by 2% agarose gel electrophoresis^11,12^.

### Antimicrobial susceptibility testing

According to the guidelines of the Clinical and Laboratory Standards Institute (CLSI), the antimicrobial susceptibility testing was performed for the isolates by using the micro-broth dilution method with Mueller–Hinton II broth (BBL™ Mueller–Hinton II broth, MHB, BD, USA) containing 5% fetal bovine serum (FBS)^13^. Sixteen antimicrobials were selected, including amoxicillin, cefazolin, ceftiofur, doxycycline, enrofloxacin, erythromycin, florfenicol, gentamicin, lincomycin, lincospectin, oxytetracycline, penicillin G, tiamulin, trimethoprim-sulfamethoxazole, tylosin, and tylvalosin. The concentration of the antimicrobial agents ranged from 0.0625 to 1024 μg/mL, and the minimum inhibitory concentration (MIC) breakpoint values for *Streptococcus* spp. were provided by the CLSI veterinary breakpoints^13^, European Committee for Antimicrobial Susceptibility Testing (EUCAST)^14^, Food and Drug Administration (FDA)^15^, and reported data^16^. *Escherichia coli* (ATCC 25,922), *Pseudomonas aeruginosa* (ATCC 27,853), *Enterococcus faecalis* (ATCC 29,212), *Staphylococcus aureus* (ATCC 29,213) and *Streptococcus pneumoniae* (ATCC 49,619) were used as quality control strains following CLSI guidance.

### MLST

Seven house-keeping genes, including *aroA*, *cpn60*, *dpr*, *gki*, *mutS*, *recA* and *thrA*, were amplified by PCR for the nucleic acid extracted from the purified *S. suis* isolates according to the MLST method established by King et al. and Rehm et al.^3,17^. The confirmed PCR products were then sent to Tri-I Biotech, Inc. (Taiwan) for 5' to 3' and 3' to 5' sequencing using an Applied Biosystems 3730 DNA Analyzer (Applied Biosystems, USA). Subsequently, the sequencing results were uploaded to BioNumerics® version7.6.3 (Applied Maths, USA), and the obtained alleles and STs were compared with the PubMLST database. New allele sequences and STs were uploaded to PubMLST to obtain the allele profile and define the genotypes^18^. By using the eBURST analysis of PubMLST, the *S. suis* isolates with respective allele profiles were clustered according to the association of STs. When 6 of 7 alleles among the *S. suis* isolates were identical, these isolates were identified as the same cluster. The isolates that did not belong to any cluster were singletons^3^. The minimum spanning tree (MST) was calculated using BioNumerics® version 7.6 according to the unweighted pairgroup method with an arithmetic mean algorithm (UPGMA). In order to understand the general distribution of the *S. suis* isolates, MST was set as distance ≤ 1 for partitioning, in which the involving nodes were clustered as CCs^19^.

### Statistical analysis

Association of isolation sites, serotypes, and antimicrobial drug resistance were analyzed by Chi-square test and Fisher’s exact test, depending on the number of samples. SPSS was used for statistical analysis and *p* value < 0.05 was considered to be statistically significant. If *p* value < 0.01, there was an extremely significant association.

## Results

### Bacterial isolation, identification, and serotyping

This study identified serotypes of 388 isolates collected from 355 diseased pigs (Table 1). The major serotypes were serotypes 3 (79/388, 20.4%), 7 (12.9%), 8 (11.6%), 2 and 9 (8.2%), and 1 (7.2%), followed by serotypes 4, 5, 1/2 and 21. The serotypes of 73 isolates (18.8%) could not be identified by PCR. The main isolation sites were shown in Table 1. Among the 355 diseased pigs, single serotype was isolated from 348 pigs (348/355, 98.0%), while two serotypes were isolated from seven pigs (7/355, 2.0%). Among the seven pigs, five pigs had one strain with an unknown serotype; and for the other strain, there were 3 pigs with serotypes 4, 7, and 8 individually and 2 pigs with serotype 9. The remaining two of the seven pigs were serotypes 3 and 8, and serotypes 7 and 8, respectively.

**Table 1:** Serotype distribution in various feeding stages and organs.

Category	Serotype 1	Serotype 2	Serotype 3	Serotype 7	Serotype 8	Serotype 9	N	OT	Total
	(*n* = 28)	(*n* = 32)	(*n* = 79)	(*n* = 50)	(*n* = 45)	(*n* = 32)	(*n* = 73)	(*n* = 49)	(*n* = 388)
Pig production stages									
Suckling pigs	27**		7	7	1	1	10	11	64
Nursery pigs	1	16	52	36	38**	24	50	28	245
Growing pigs		8*	13	6	5	5	6	7	50
Fattening pigs		8**	5			1	3	2	19
Breeding pigs				1		1	2		4
Unknow			2		1		2	1	6
Organ									
Brain/cerebrospinal fluid	10**	1	5	4	1	12**	2	7	42
Lung/Bronchus	3	29**	63*	35	37	12	56	35	270
Liver	4	1	4	5	3	3	11	5	36
Synovial fluid	9**		5	3	2	2	1	2	24
Other^#^	2	1	2	3	2	3	3		16

^**#**^includes heart, pericardial fluid, pleural fibrin, spleen, stomach, eye and nasal.

N: Unidentified serotype, OT: Other serotypes include 1/2, 4, 5, 12, 14, 16, 17, 18, 19, 21, 24, 27, 29, 31.

*p < 0.05; **p < 0.01.

Serotype 1 was frequently isolated from brains, cerebrospinal fluid or synovial fluid at the suckling stage (*p* < 0.01). Serotypes 3, 7, 8, and 9 were mostly isolated from lungs or bronchial lumina at the nursing stage. In addition, serotype 2 was most likely isolated from lungs at the growing-finishing stage (*p* < 0.05) (Table 1).

### Antimicrobial susceptibility testing

**Figure 1:**
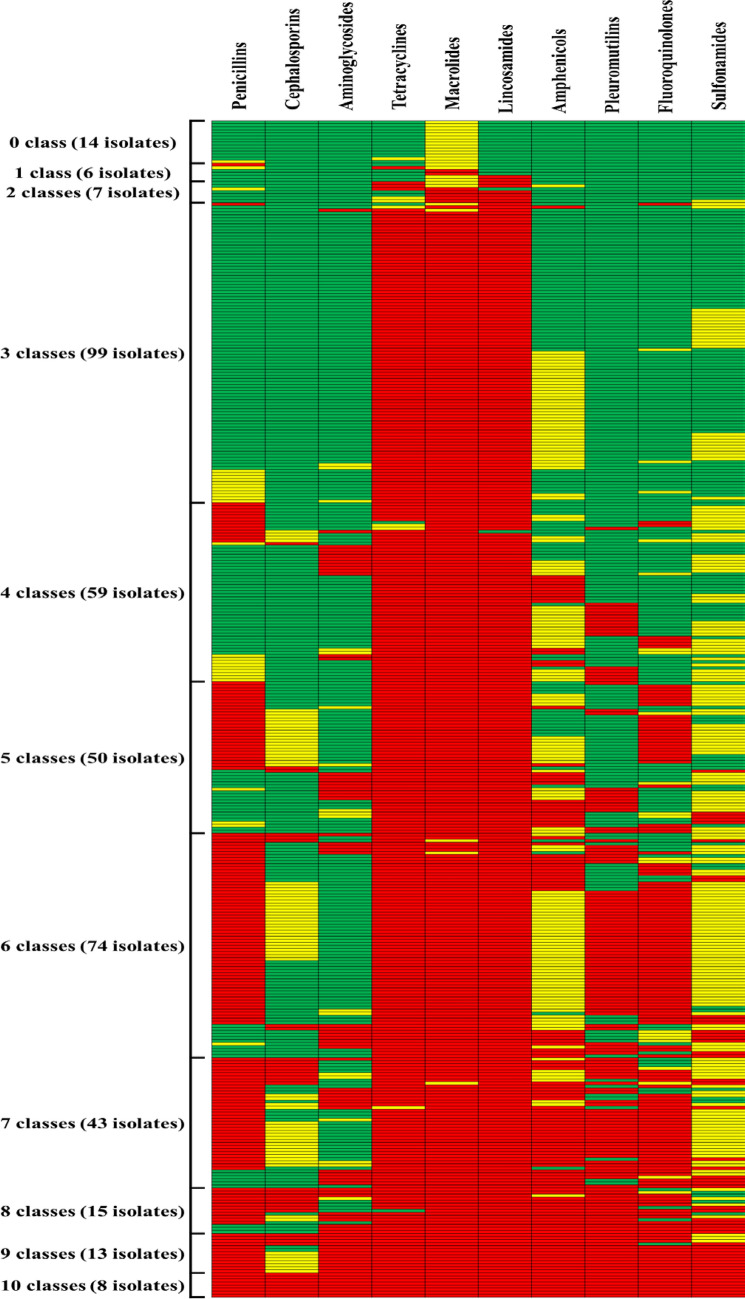
Heatmap showing antimicrobial susceptibility profiles of *S. suis* isolates. Rows represent bacterial isolates and columns represent antibiotics. The blocks indicate antibiotic susceptibility (green: susceptible, yellow: intermediate, red: resistant). The heatmap was generated by using Microsoft Excel 2010.

Antimicrobial susceptibility testing was performed on all 388 isolates by using the broth microdilution method according to the CLSI standards. The results showed that *S. suis* was highly susceptible to ceftiofur (96.4%), cefazolin (91.0%), trimethoprim /sulfamethoxazole (86.9%) and gentamicin (79.6%). The isolates showed moderate susceptibility (40–70%) to florfenicol (68.8%), amoxicillin (61.1%), enrofloxacin (60.1%), tiamulin (60.1%), penicillin G (58.0%) and doxycycline (54.9%). The susceptibility to lincospectin (33.2%), erythromycin (14.7%), tylosin (8.8%), oxytetracycline (8.0%) and lincomycin (5.2%) was lower than 40% (Table 2). Notably, most *S. suis* isolates showed low susceptibilities (< 10%) to tylosin, oxytetracycline and lincomycin in this study. Since there was no clinical breakpoint of *S. suis* for tylvalosin, the results were shown as MIC_50_ and MIC_90_, which were 256 and 1,024 μg/mL, respectively. The pattern of antibiotic resistance was analyzed according to the resistance of *S. suis* to different drug classes. Only 14 isolates (14/388, 3.6%) were susceptible to all classes of antimicrobial agents, 112 isolates (112/388, 28.9%) were resistant to 1–3 drug classes, 184 isolates (184/388, 47.4%) were resistant to 4–6 drug classes, 78 isolates (78/388, 20.1%) were resistant to more than seven drug classes, and 8 isolates were resistant to all classes of drugs. The most common antimicrobial resistance pattern was the resistance to tetracyclines, macrolides and lincosamides, which was found in 89.9% (349/388) of the *S. suis* isolates. (Fig. 1).

**Table 2:**
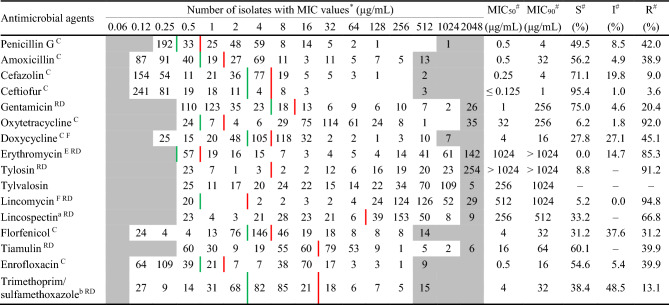
MIC distribution of *S. suis* isolates (*n* = 388).

^*^The white area indicates the dilution range of the antimicrobials which were tested on the isolates. Green and red vertical lines indicate respectively the susceptible and resistant clinical breakpoints recommended by the CLSI(^C^), EUCAST(^E^), FDA(^F^) and reported data(^RD^).

^#^MIC_50_: The MIC that inhibits 50% of the tested isolates, MIC_90_: The MIC that inhibits 90% of the tested isolates.

S: Susceptible percentage, I: Intermediate percentage, R: Resistant percentage.

^a^Lincospectin combination was tested in a concentration ratio of 1:2. MIC values in the table represent spectinomycin concentrations.

^b^Trimethoprim/sulfamethoxazole combination was tested in a concentration ratio of 1:5. MIC values in the table represent sulfamethoxazole concentrations.

The relation between the antimicrobial susceptibility and the serotypes indicated that serotype 1 was highly susceptible to penicillin G, amoxicillin, cefazolin, ceftiofur, tiamulin and enrofloxacin. Serotypes 2 and 3 also had high susceptibility to penicillin G, amoxicillin, cefazolin, ceftiofur, enrofloxacin, tiamulin; and were additionally highly susceptible to gentamicin, florfenciol and trimethoprim/sulfamethoxazole. Serotype 7 had better susceptibility only to cefazolin, ceftiofur and trimethoprim/sulfamethoxazole. Serotypes 8 and 9 were highly susceptible to cefazolin, ceftiofur, gentamicin and trimethoprim/sulfamethoxazole (Table S1).

### Multilocus sequence typing (MLST)

**Figure 2:**
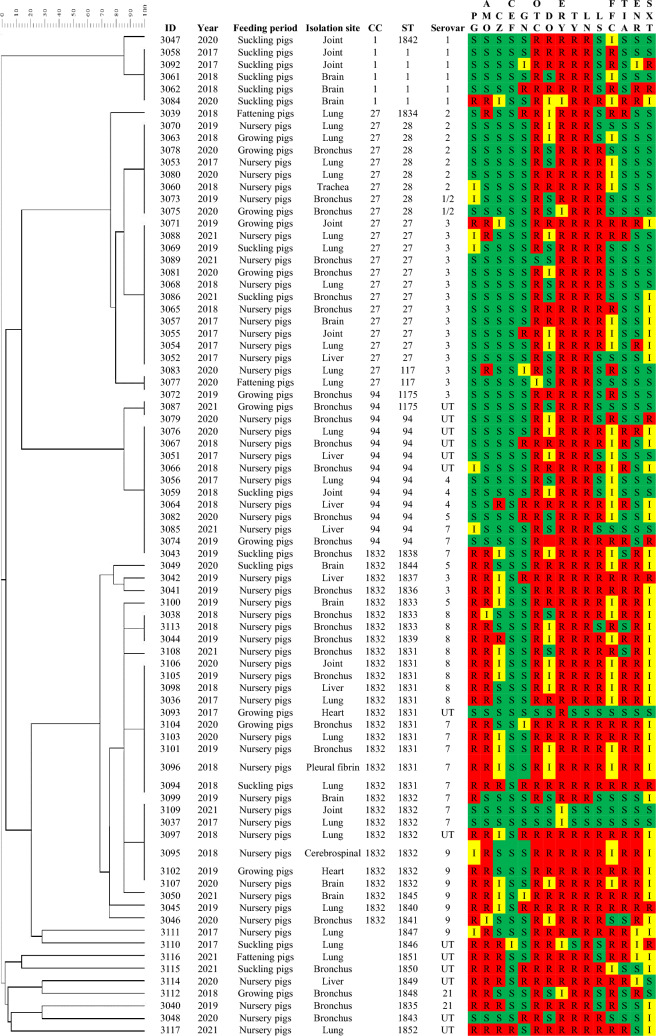
Phylogenetic dendrogram constructed from the ST profiles of *S. suis* isolates. PG: Penicillin G, AMO: Amoxicillin, CZ: Cefazolin, CEF: Ceftiofur, GN: Gentamicin, OTC: Oxytetracycline, DO: Doxycycline, ERY: Erythromycin, TY: Tylosin, LN: Lincomycin, LS: Lincospectin, FFC: Florfenicol, TIA: Tiamulin, ENR: Enrofloxacin, SXT: Sulfamethoxazole-trimethoprim. The heatmap was generated by using Microsoft Excel 2010.

**Figure 3:**
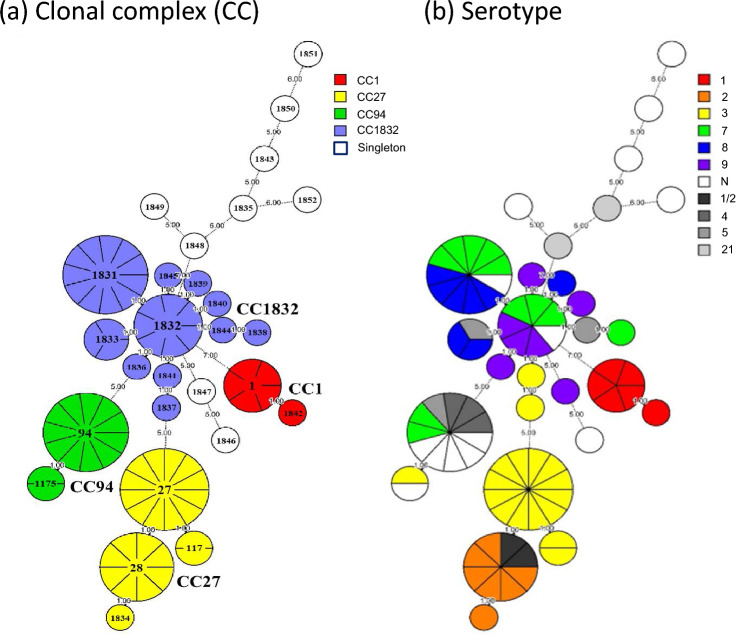
The MST graph constructed from the ST profiles of *S. suis* isolates (*n* = 80). (**a**) The relationship between CC and serotypes of *S. suis* isolates was characterized. The nodes were labeled based on the ST data, and the branch lengths were shown according to the discrepancy of allele profile between connected nodes. (**b**) The relationship between serotypes and STs.

Eighty *S. suis* clinical isolates in numbers proportional to their serotypes were selected for the MLST, which included serotypes 1 (n = 6), 1/2 (n = 2), 2 (n = 7), 3 (n = 17), 4 (n = 3), 5 (n = 3), 7 (n = 11), 8 (n = 8), 9 (n = 7), 21 (n = 2), and 14 unidentified isolates. Seventeen new alleles were found out of 7 pairs of house-keeping genes, including 5 alleles of *aroA* gene, 8 alleles of *cpn60* gene, 2 alleles of *dpr* gene, and 1 allele of *gki* gene and *recA* gene. New sequences of house-keeping gene were submitted to PubMLST for verification and the allele profiles of the new sequences were then registered to PubMLST to define the 22 new STs discovered in this study. The result of the 80 *S. suis* isolates showed that *aroA*, *cpn60*, *gki*, *dpr*, *mutS*, *recA* and *thrA* genes exerted respectively 16, 15, 13, 9, 11, 9 and 12 different alleles, forming 28 different STs. ST27 (12/80, 15.0%), ST94 (13.8%) and ST1831 (13.8%) were the major STs, followed by ST28 (10.0%), ST1832 (8.7%), ST1 (6.3%), ST1833 (3.7%), ST117 (2.5%) and ST1175 (2.5%) (Fig. 2). The 80 *S. suis* isolates were divided into 4 clusters and 9 singletons. As depicted in the phylogenetic dendrogram and MST, 28 STs were divided into 4 CCs. CC1 was composed of 6 *S. suis* isolates and 2 STs. CC27 was composed of 23 *S. suis* isolates and 4 STs, and ST27 was predicted as the ancestral type of this cluster. CC94 was composed of 13 *S. suis* isolates and 2 STs. CC1832 was composed of 29 *S. suis* isolates and 11 STs, and ST1832 was predicted as the ancestral type of this cluster (Figs. 2 and 3a).

Further analysis of the association between serotypes and STs among the 80 *S. suis* isolates revealed that most of the isolates identified as serotypes 1, 2 and 3 belonged to ST1, ST28 and ST27, respectively. Both serotypes 2 and 3 were categorized in CC27. In addition, isolates identified as serotypes 7, 8 and 9 were mostly ST1831 and ST1832, which belonged to CC1832 (Table 3 and Fig. 3b). Taken together, specific STs may be related to serotypes.

**Table 3:** Serotype distribution among sequence types (STs).

Category	Serotype 1	Serotype 2	Serotype 3	Serotype 7	Serotype 8	Serotype 9	N	OT	Total
	(*n* = 6)	(*n* = 7)	(*n* = 17)	(*n* = 11)	(*n* = 8)	(*n* = 7)	(*n* = 14)	(*n* = 10)	(*n* = 80)
Sequence type (ST)									
ST1	5								5
ST27			12						12
ST28		6						2	8
ST94				2			5	4	11
ST1831				5	5		1		11
ST1832				3		3	1		7
Other^#^	1	1	5	1	3	4	7	4	26

*#*includes ST117, ST1175, and ST1833 to ST1852.

N: Unidentified serotype, OT: Other serotypes include 1/2, 4, 5, 21.

## Discussion

In this study, *S. suis* isolates were mostly serotypes 3, 7 and 8, followed by serotypes 1, 2 and 9. In comparison with the serotype distribution in other countries, serotype 2 is the most prevalent in China, Japan, Vietnam, Thailand, Spain, Italy, France, Poland and White Russia^20^. The second prevalent serotype varies with countries, which are mostly serotypes 3 and 4 in Korea, serotypes 3, 1/2 and 7 in the U.S., serotype 9 in the Netherlands, and serotypes 1 and 14 in Britain^21,22^. Our result is close to the data reported in the U.S., in which serotypes 3 and 7 are more prevalent. In addition, our results are also in agreement with previous finding that *S. suis* isolated from diseased pigs was mainly composed of limited serotypes^23^. In general, the *cps* genome is the key region of gene recombination, leading to *cps* transformation between strains^24^. New *cps* genomes of *S. suis* have been found successively in recent years^25^, which expands the genetic diversity and reduces the quantity of unidentified isolates. In the current study, there are 18.8% of isolates that cannot be identified using cps gene by PCR and RFLP; therefore, evaluation of the virulence potential and the effects of these likely new genomes in the pathogenesis is warranted.

As shown in Table 1, serotype 1 was frequently isolated from brains, cerebrospinal fluid or synovial fluid at the suckling stage (*p* < 0.01). Furthermore, a very significant association between the stage of suckling pigs and the strains derived from cerebrospinal fluid and synovial fluid was found (*p* < 0.001, Table S2). The phenomenon that suckling pigs were more prone to brain and joint infections may be attributed to the invasive operations such as ear notching, tail cutting, teeth clipping, castration and drug injection during the lactation stage, leading to exposure of opportunistic bacteria in the environment such as *S. suis*. In addition, significant association between the strains isolated from respiratory tract and the stages of growing and finishing pigs was identified. *S. suis* that colonizes in healthy adult pigs rarely causes symptoms; however, suckling pigs may be prone to get infected from the healthy adult *S. suis* carriers through cross-fostering^26^.

The antimicrobial susceptibility testing showed that the isolates were highly susceptible to ceftiofur, cefazolin, trimethoprim/sulfamethoxazole and gentamicin, and moderately susceptible to florfenicol, amoxicillin, enrofloxacin, tiamulin, penicillin G and doxycycline. By applying the PK/PD indices that are calculated by the antimicrobial susceptibility data (Fig. S1) in combination with pharmacokinetic parameters of the drugs, effective treatment can be reasonably recommended. For instance, the suckling pigs were prone to be infected with serotype 1 which mainly causes suppurative meningitis or/and arthritis, so amoxicillin, ceftiofur and tiamulin were recommended for treatment. The pigs at the nursing stage were especially susceptible to serotypes 3, 7, 8 and 9, in which the infection in the respiratory system was relatively frequent. Considering the pharmacokinetic characteristics, cefazolin, ceftiofur, or trimethoprim/sulfamethoxazole was recommended for treatment. Among them, β-lactams are time-dependent bactericidal antimicrobial agents. In this study, the MIC_90_ of *S. suis* serotype 3, 7, 8 and 9 for ceftiofur were 0.25, 2, 0.5, and 0.25 μg/mL, respectively. After administration of ceftiofur at 5 mg/kg, AUC_0-24 h_ was calculated as approximately 358.84 μg•h/mL^27^. The index of the AUC/MIC ratio was used to describe the antibacterial activity of time-dependent  drugs. An AUC/MIC ratio > 125 h is generally considered good antimicrobial activity^28,29^. As AUC/MIC_90_ calculated for serotype 3, 7, 8 and 9 for ceftiofur ranged from 179.42–1435.36 h (> 125 h), indicating that this antimicrobial agent could be recommended for treating these infection of the four *S. suis* serotypes. Similar in rationale, the *S. suis* infection of pigs at the growing-finishing stage was mainly serotype 2, which was most likely to be isolated from the respiratory system but may cause systemic infection. The MIC values of penicillin G (MIC_90_ = 0.5 μg/mL), amoxicillin (MIC_90_ = 0.5 μg/mL), cefazolin (MIC_90_ = 0.25 μg/mL) and ceftiofur (MIC_90_ = 0.25 μg/mL) were obtained. When these data were combined with the pharmacokinetic information, oral administration of amoxicillin at 20 mg/kg or injection of ceftiofur at 5 mg/kg can give rise to AUC/MIC_90_ ≥ 125 h, and thus could be advised to treat serotype 2 infections. Notably, the parameters might be different for different drugs, organisms or bacteria^30^. In addition, it should be noted that healthy asymptomatic pigs are the source of human *S. suis* infection, in which most of the strains are serotype 2 and 90.2% of the cases are in Asia. Therefore, more attention should be paid to *S. suis* serotype 2 strains persisting in finishing pigs^31^.

Resistance of *S. suis* to tetracycline is prevalent worldwide. High proportion of resistance has been reported in the Americas (Canada 80–90%, Brazil 98%)^32,33^, Asia (China 99%, Korea 98%, Japan 78–100%, Thailand 96%, Vietnam 100%)^34–38^ and Spain (95%)^39^. Some of the European countries show a moderate degree of drug resistance (40.3–73.3%), and Sweden shows the lowest degree (7.7%)^20^. The resistance to erythromycin is higher in Taiwan (87%), South Korea (96%) %), the United States (82%) and Australia (99%), and lower in China (68%) and Japan (55–66%). Although β-lactams have been frequently used in pigs in recent years, most *S suis* strains are still sensitive to them and maintain a low drug resistance. The resistance rates to penicillin and ampicillin are 0–27% and 0–23%, respectively.

The proportion of *S suis* resistant to macrolides and lincosamides is rising globally^20^. According to the results of the drug resistance the results of the drug resistance, it can be seen that 89.9% of *S. suis* isolates were resistant to three classes of drugs including tetracyclines, macrolides and lincosamides. Li et al. have reported that *S. suis* resistant to chloramphenicols, macrolides, lincosamides, chloramphenicols, fluoroquinolones, aminoglycosides and hydantoins is the most common drug resistance profile in China^40^. In addition, *S. suis* strains become resistant to five classes of antimicrobials including tetracyclines, lincosamides, fluoroquinolones, sulfonamides and hydantoins in Brazil^33^. The percentage of multi-drug resistance (resistance to more than 3 classes of antimicrobials) of the isolates collected in this study was as high as 93.0%, and 39.4% of the isolates were resistant to more than 6 classes of antimicrobials. Our result is similar to the data from Li et al. (2012) and Soares et al. (2014), which showed the proportion of *S. suis* isolates resistant to more than 3 classes (98.7%, 99.6%) and 6 classes (35.9%, 85.0%) of antimicrobials in China and Brazil^33,40^.

Comparing the MLST result with serotypes, it was apparent that the pigs at the suckling stage were mainly infected with *S. suis* serotype 1, which corresponded to ST1 (*n* = 5) and CC1. *S. suis* serotype 2 mainly corresponded to ST28 (n = 6) (Table 3). Goyette-Desjardins et al*.* found that most of the invasive strains with high virulence belong to CC1, including ST1, ST6, ST7 and ST11, which are usually related to septicemia, meningitis and arthritis^21^. The current study showed that *S. suis* isolated from the diseased pigs with central nervous system symptoms and arthritis also belonged to CC1. In contrast, CC27 might be related more to the respiratory tract infections^3^. *S. suis* serotype 2 isolated from the growing-finishing pigs was ST28 belonging to CC27 and mostly isolated from the respiratory system. This result is similar to that of China, Japan, and the U.S., but the pathogenicity needs further investigation^21,41^. *S. suis* serotype 3 isolated from the pigs at the nursing stage was mainly ST27 of CC27 (n = 12) from respiratory systems (79.7%; 63/79). In comparison, *S. suis* serotype 8 was prone to be isolated from the pigs at the nursing stage (*p* < 0.01), and it was mostly isolated from lungs or bronchial lumina (82.2%; 37/45). These isolates were mainly ST1831 of CC1832 (n = 5). These results correspond well to previous findings that serotypes 3 and 8 are mostly restricted to lung infection^42^. CC94 included ST94 (n = 11) and ST1175 (n = 2), which corresponded to the unidentified *S. suis* isolates (n = 6), serotypes 4 (n = 3), 7 (n = 2), 3 (n=1) and 5 (n = 1) (Fig. 2). ST94 and CC94 are widely detected in the U.S., and they are the fourth most frequently seen ST and CC in North America. They are related to the pathogenic strains of serotypes 3, 4, 5, 7 and 24, but unrelated to serotypes 1, 2 and 14^43^. ST94 detected in Europe and Asia is mainly related to serotypes 4, 16 and unidentified strains^44^. Taken together, different serotypes exerting distinct infectious efficiency might determine the virulence or geographical distribution of *S. suis*^45^.

CC1832 is the largest CC in this study. It consisted of ST1831, ST1832, ST1833, ST1836, ST1837, ST1838, ST1840, ST1841 and ST1844, which corresponded mostly to serotypes 7, 8 and 9 (*n* = 23) and were mostly isolated from the pigs at the nursing stage (*n* = 23). This CC has a higher proportion of drug resistance to penicillin G, amoxicillin, cefazolin, ceftiofur, tiamulin and enrofloxacin than the other CCs. The pigs at this stage are susceptible to viral infections, such as PRRSV and PCV2, which are likely to cause opportunistic/secondary bacterial respiratory tract infections, e.g., *Glaesserella parasuis*, *Mycoplasma hyorhinis*, *Bordetella bronchiseptica*, and *S. suis*^46,47^. The antimicrobial agents are frequently used for pigs at this stage, leading to a relatively high drug resistance to common *S. suis* serotypes. The *S. suis* isolated from diseased pigs in this study was mostly from the respiratory system, and most of the isolates showed multi-drug resistance. Therefore, clinical use of antimicrobial agents should be selected with caution based on the characteristics of clinical isolates and the results of antimicrobial drug susceptibility.

## Conclusion

*S. suis* is an important bacterial zoonotic pathogen with a high risk of occupational infection. In this study, aside from newly identified STs of *S. suis* strains, STs and serotypes were found to exert certain association, which can be further related to the feeding stages of pigs and the antimicrobial drug susceptibility. This is the first study that reports the serotype distribution, bacterial resistance and molecular epidemiological analysis of *S. suis* isolated from diseased pigs at different feeding stages in Taiwan. The epidemiological investigation contributes to a better understanding of the role of this bacteria and more proper treatment strategies.

## Supplementary Information

Supplementary Information.

## Data Availability

The datasets generated in the current study are available in the PubMLST, web link [https://pubmlst.org/bigsdb?db=pubmlst_ssuis_isolates&page=query&prov_field1=f_country&prov_value1=Taiwan&submit=1].
