# Supplementary material for: Serotype and multilocus sequence typing of Streptococcus suis from diseased pigs in Taiwan
Source: Sci Rep. 2023 May 22;13:8263. doi: 10.1038/s41598-023-33778-9 (PMC10202909; doi:10.1038/s41598-023-33778-9)
Supplement: Supplementary file 1 — Supplementary Information. [file 41598_2023_33778_MOESM1_ESM.docx]

Table S1 The number and percentage (%) of antimicrobial resistant isolates in different serotypes of *S. suis*.

| **Antimicrobials** | **Serotype 1** | | | **Serotype 2** | | | **Serotype 3** | | | **Serotype 7** | | | **Serotype 8** | | | **Serotype 9** | | | **Untypable** | | | **Other** | | **Total** | |
| --- | --- | --- | --- | --- | --- | --- | --- | --- | --- | --- | --- | --- | --- | --- | --- | --- | --- | --- | --- | --- | --- | --- | --- | --- | --- |
|  | (*n* = 28) | | | (*n* = 32) | | | (*n* = 79) | | | (*n* = 50) | | | (*n* = 45) | | | (*n* = 32) | | | (*n* = 73) | | | (*n* = 49) | | (*n* = 388) | |
|  | No. | | R% | No. | | R% | No. | | R% | No. | | R% | No. | | R% | No. | | R% | No. | | R% | No. | R% | No. | R% |
| Penicillin G | 1 |  | 3.6 | 1 |  | 3.1 | 7 |  | 8.9 | 28 |  | 56.0 | 37 |  | 82.2 | 26 |  | 81.3 | 43 |  | 58.9 | 20 | 40.8 | 163 | 42.0 |
| Amoxicillin | 1 |  | 3.6 | 2 |  | 6.3 | 12 |  | 15.2 | 19 |  | 38.0 | 36 |  | 80.0 | 26 |  | 81.3 | 35 |  | 47.9 | 20 | 40.8 | 151 | 38.9 |
| Cefazolin | 0 |  | 0.0 | 0 |  | 0.0 | 2 |  | 2.5 | 7 |  | 14.0 | 2 |  | 4.4 | 1 |  | 3.1 | 18 |  | 24.7 | 5 | 10.2 | 35 | 9.0 |
| Ceftiofur | 0 |  | 0.0 | 0 |  | 0.0 | 0 |  | 0.0 | 3 |  | 6.0 | 1 |  | 2.2 | 0 |  | 0.0 | 9 |  | 12.3 | 1 | 2.0 | 14 | 3.6 |
| Vancomycin | 0 |  | 0.0 | 0 |  | 0.0 | 0 |  | 0.0 | 0 |  | 0.0 | 1 |  | 2.2 | 0 |  | 0.0 | 0 |  | 0.0 | 0 | 0.0 | 1 | 0.3 |
| Gentamicin | 9 |  | 32.1 | 5 |  | 15.6 | 13 |  | 16.5 | 14 |  | 28.0 | 3 |  | 6.7 | 5 |  | 15.6 | 21 |  | 28.8 | 9 | 18.4 | 79 | 20.4 |
| Oxytetracycline | 28 |  | 100.0 | 31 |  | 96.9 | 75 |  | 94.9 | 42 |  | 84.0 | 38 |  | 84.4 | 29 |  | 90.6 | 65 |  | 89.0 | 49 | 100.0 | 357 | 92.0 |
| Doxycycline | 22 |  | 78.6 | 16 |  | 50.0 | 20 |  | 25.3 | 16 |  | 32.0 | 15 |  | 33.3 | 21 |  | 65.6 | 40 |  | 54.8 | 25 | 51.0 | 175 | 45.1 |
| Erythromycin | 24 |  | 85.7 | 25 |  | 78.1 | 78 |  | 98.7 | 35 |  | 70.0 | 39 |  | 86.7 | 27 |  | 84.4 | 59 |  | 80.8 | 44 | 89.8 | 331 | 85.3 |
| Clarithromycin | 25 |  | 89.3 | 20 |  | 62.5 | 71 |  | 89.9 | 32 |  | 64.0 | 36 |  | 80.0 | 24 |  | 75.0 | 51 |  | 69.9 | 38 | 77.6 | 297 | 76.5 |
| Tylosin | 28 |  | 100.0 | 28 |  | 87.5 | 78 |  | 98.7 | 40 |  | 80.0 | 38 |  | 84.4 | 29 |  | 90.6 | 66 |  | 90.4 | 47 | 95.9 | 354 | 91.2 |
| Lincomycin | 28 |  | 100.0 | 31 |  | 96.9 | 78 |  | 98.7 | 41 |  | 82.0 | 40 |  | 88.9 | 31 |  | 96.9 | 70 |  | 95.9 | 49 | 100.0 | 368 | 94.8 |
| Lincospectin | 6 |  | 21.4 | 20 |  | 62.5 | 62 |  | 78.5 | 29 |  | 58.0 | 35 |  | 77.8 | 28 |  | 87.5 | 45 |  | 61.6 | 34 | 69.4 | 259 | 66.8 |
| Florfenicol | 13 |  | 46.4 | 4 |  | 12.5 | 15 |  | 19.0 | 17 |  | 34.0 | 12 |  | 26.7 | 11 |  | 34.4 | 35 |  | 47.9 | 14 | 28.6 | 121 | 31.2 |
| Tiamulin | 3 |  | 10.7 | 4 |  | 12.5 | 16 |  | 20.3 | 27 |  | 54.0 | 23 |  | 51.1 | 24 |  | 75.0 | 40 |  | 54.8 | 18 | 36.7 | 155 | 39.9 |
| Enrofloxacin | 4 |  | 14.3 | 5 |  | 15.6 | 11 |  | 13.9 | 21 |  | 42.0 | 35 |  | 77.8 | 28 |  | 87.5 | 29 |  | 39.7 | 22 | 44.9 | 155 | 39.9 |
| Sulfamethoxazole-  trimethoprim | 9 |  | 32.1 | 2 |  | 6.3 | 3 |  | 3.8 | 7 |  | 14.0 | 3 |  | 6.7 | 6 |  | 18.8 | 17 |  | 23.3 | 4 | 8.2 | 51 | 13.1 |

**No.**: number of isolates; **R%**: percentage of antimicrobial resistant isolates

**Table S2** Organ distribution among feeding periods.

| **Feeding period** | **Brain/ Spinal cord** | | **Lung/ Bronchus** | | **Liver** | | **Joint** | | **Other^#^** | | **Total** |
| --- | --- | --- | --- | --- | --- | --- | --- | --- | --- | --- | --- |
|  | (*n* = 42) | | (*n* = 270) | | (*n* = 36) | | (*n* = 24) | | (*n* = 16) | | (*n* = 388) |
| Suckling pigs | 15 | *** | 23 | *** | 9 |  | 12 | *** | 5 |  | 64 |
| Nursery pigs | 26 |  | 178 |  | 26 |  | 11 |  | 4 |  | 245 |
| Growing pigs |  |  | 43 | ** | 1 |  | 1 |  | 5 |  | 50 |
| Fattening pigs | 1 |  | 18 | * |  |  |  |  |  |  | 19 |
| Breeding pigs |  |  | 2 |  |  |  |  |  | 2 |  | 4 |
| Unknow |  |  | 6 |  |  |  |  |  |  |  | 6 |

**^#^**: include heart, pericardial fluid, pleural fibrin, spleen, stomach, eye, and nasal.

**No.**: number of isolates

*: *p* < 0.05; **: *p* < 0.01; ***: *p* < 0.001

**
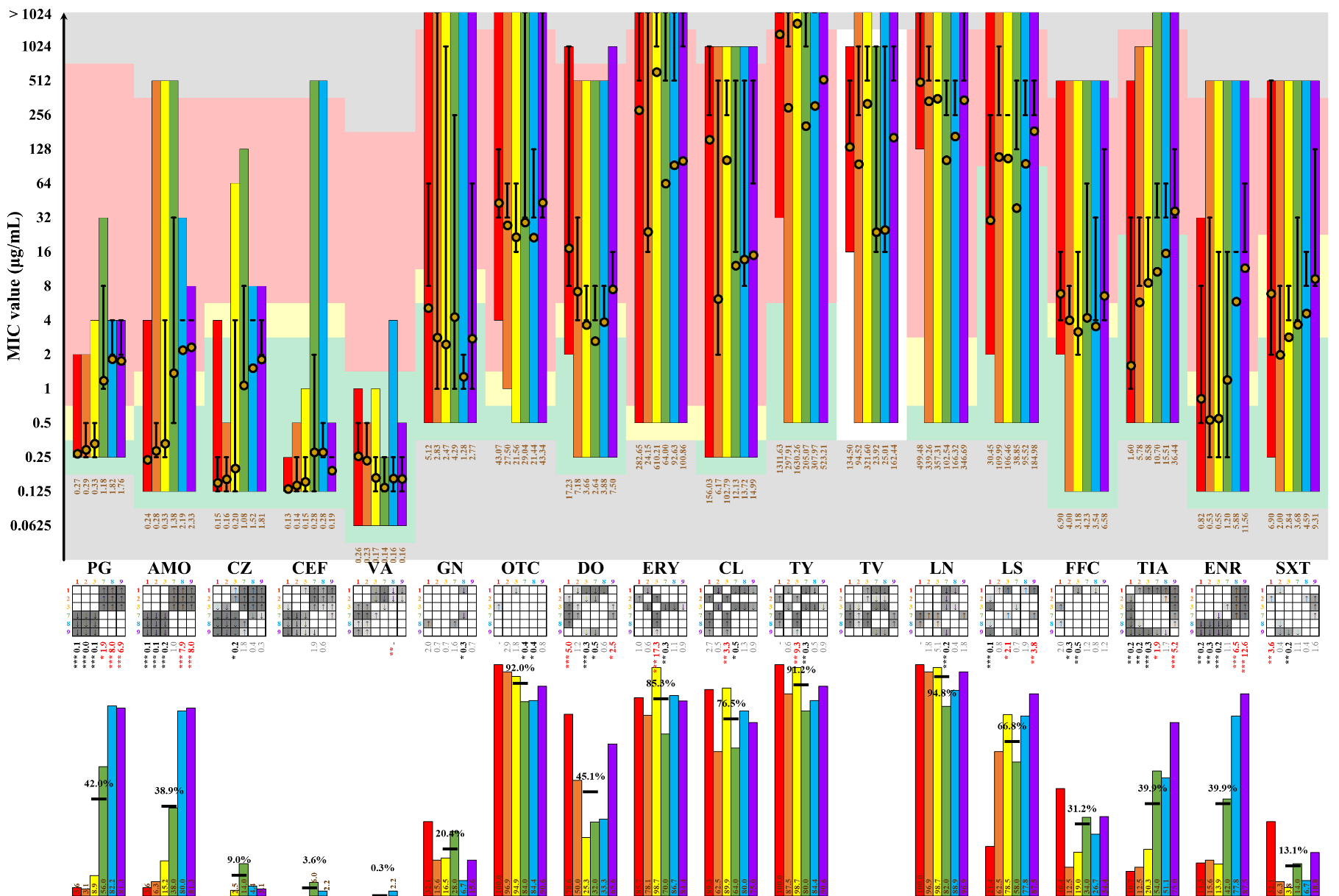

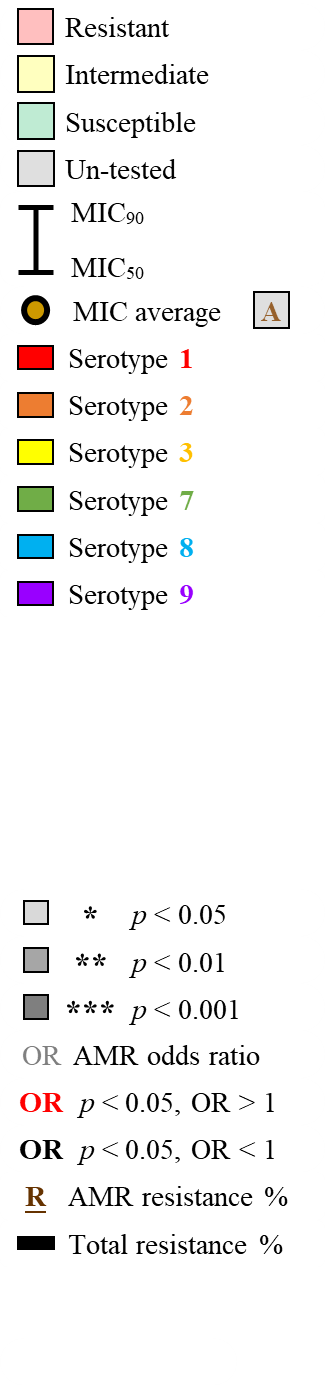
**

**Figure S1** MIC values of antimicrobials in different serotypes of *S. suis*.
